# Primary tumour iodine avidity in relation to uptake in persistent metastatic disease in papillary and poorly differentiated thyroid cancer

**DOI:** 10.1007/s12020-023-03414-7

**Published:** 2023-06-07

**Authors:** Joachim N. Nilsson, Per Grybäck, C. Christofer Juhlin, Christel Hedman, Catharina Ihre Lundgren

**Affiliations:** 1 Department of Molecular Medicine and Surgery, Karolinska Institutet, Stockholm, Sweden; 2 Department of Medical Radiation Physics and Nuclear Medicine, Karolinska University Hospital, Stockholm, Sweden; 3 Department of Oncology-Pathology, Karolinska Institutet, Stockholm, Sweden; 4 Department of Pathology and Cancer Diagnostics, Karolinska University Hospital, Stockholm, Sweden; 5 Stockholms Sjukhem Foundation’s Research and Development Department, Stockholm, Sweden; 6 Division of Palliative Care, Department of Clinical Sciences, Lund University, Lund, Sweden; 7 Department of Breast, Endocrine Tumours and Sarcoma, Karolinska University Hospital, Stockholm, Sweden

**Keywords:** Radioiodine therapy, Iodine avidity, Papillary thyroid cancer, Poorly differentiated thyroid cancer

## Abstract

**Purpose:**

Patients with persistent or recurrent papillary and poorly differentiated thyroid cancer can be effectively treated with radioiodine, if the tumour tissue is iodine-avid. However, iodine-avidity status is often unknown at the time of initial radioiodine treatment, limiting any adaptive approach. This study aimed to clarify the relationship between pre-therapeutic iodine avidity in primary tumour tissue, initial lymph node metastases and iodine uptake in subsequent metastases.

**Methods:**

Iodine avidity was prospectively assessed pre-therapeutically in 35 patients by injection of tracer amounts of iodine-131 two days prior to surgery. Iodine concentrations in resected tissue samples were measured, enabling accurate and histologically verifiable iodine avidity data for both primary tumour and initial lymph node metastases. Iodine uptake in persistent metastatic disease was assessed by review of radiology, and treatment response was examined through journal studies.

**Results:**

Out of data from 35 patients, 10 had persistent disease at presentation or during follow-up (range 19–46 months). Four patients had non-avid persistent metastatic disease, all with low iodine avidity in their primary tumours and initial lymph node metastases. Patients with low pre-therapeutic iodine avidity did not appear to have greater risk of persistent disease.

**Conclusion:**

The results indicate a close link between pre-therapeutically measured iodine concentrations in primary tumours with iodine avidity of any subsequent metastases.

## Introduction

Patients with papillary thyroid cancer (PTC) have excellent overall survival, due to the indolent nature of most tumours [1, 2]. Treatment consists primarily of thyroidectomy, lymph node resection, radioiodine (RAI) therapy and TSH suppression [3]. There has been a trend in the last decades of less use of RAI and TSH suppression for patients at lower risks of recurrence. However, a small minority of PTC patients will suffer from progressive disease with distant metastases [4]. The incidence of PTC has been increasing over the last few decades (albeit mostly consisting of low-risk tumours), highlighting the need for refined selection of treatment, adaptation and avoidance of overtreatment [5, 6]. Poorly differentiated thyroid cancer (PDTC) comprises 2–15% of all thyroid cancers, has worse prognosis than PTC and frequently progresses into RAI refractory disease, requiring more aggressive treatment [7, 8]. Both PTC and PDTC has a recurrence rate of 10–30% which persists over many years, requiring long-term follow-up [9–11].

Iodine avidity of metastases is known to be correlated to patient outcome [4], but not all patients with visible iodine uptake in distant metastases will have durable structural response to radioiodine treatment, possibly due to present-but-inadequate avidity or too low activity administrations [12]. Because of this, information on which patients are expected to harbour or develop non-avid metastases could guide RAI treatment. Dosage could be adapted to the avidity of the tumour, potentially avoiding ineffective treatments and improving efficacy for high-risk patients with iodine-avid tumours. The material has previously been studied to find parameters correlated to iodine avidity at diagnosis, indicating strong links between high thyroglobulin (Tg) expression, low Ki-67 index and high avidity in the primary tumour and initial lymph node metastases [13]. However, there is still a lack of knowledge on how relevant and reliable initial avidity is in predicting the iodine uptake status of subsequent metastases. In the present study we quantified iodine avidity as accurately as possible, by measuring iodine-131 concentrations accumulated in tumour tissue from a low activity injection prior to surgery, and compared it to the uptake of any subsequent metastases.

## Materials and methods

### Patient Inclusion

A prospective study on patients referred for PTC to Karolinska University Hospital, Stockholm, Sweden (a tertiary medical centre with a catchment area population of 2.5 million) between February 2019 and April 2021 was conducted. Patients were queried for study participation upon their referral visit and gave written informed consent before study inclusion. In total, 45 patients were included; the inclusion rate was estimated to 40% of all queried patients; the 45 patients constitute an approximate 15% of all patients referred to our centre during the study period.

Inclusion criteria were: cytologically confirmed PTC with cytological Bethesda V or VI (patients stayed included if the final histological diagnosis was PDTC). Exclusion criteria were: tumours smaller than 1 cm (as assessed by ultrasound examination), age <18 years, pregnancy, (eGFR <30 ml/min/1.7 m^2^) and difficulties understanding the study information. Included patients were subsequently excluded if there was any risk of compromising the histopathological diagnosis at pathology grossing. Follicular thyroid cancers were not included since preoperative cytology is unable to differentiate between follicular adenomas and cancers. At the time of inclusion visit, laboratory data on serum T3, T4, TSH, Tg and thyroglobulin antibodies (Tg-Ab) were collected.

### Specimen collection and measurements

Enrolled patients received an intraveneous injection of 5–10 MBq [^131^I]NaI two days prior to thyroidectomy. Two days was chosen as a likely candidate to be in the range of the effective half-life of iodine in tumour tissue, thereby limiting variance due to differences in kinetics between patients [14, 15]. A moderate iodine-restricted diet (no fish, seafood or iodinated salt) was prescribed to patients one week ahead of the injection, to limit the impact of any high-iodine intake among participants. No exogeneous or endogeneous TSH stimulation was conducted, as the participants had functioning thyroid present at the time of injection of iodine-131. After surgical removal of the thyroid gland, primary tumour and lymph nodes (removed only if pre- or perioperatively indicated), all tissue specimens were sent to pathology grossing. At grossing, samples of tumour and suspicious lymph nodes were taken for radioactive iodine quantification. If readily available, several samples of both primary tumour and lymph nodes were cut and analysed in the study. Further details of sample analysis was published previously [13]. Samples were returned to the pathology lab and fixated in formalin within a mean time of 3 h.

Tissue samples were measured in a NaI(Tl)-scintillation well chamber (Wallac, 1480 Wizard 3”). The instrument sensitivity was calibrated with known activities of iodine-131 and tuned for the 364 keV energy window (+/− 20%). The measured iodine concentrations were normalised to the injected activity (IA: the 5–10 MBq given) and the mass of the examined tissue sample (in grams) resulting in unit IA·g^−1^. Samples were corrected for competing uptake in normal thyroid tissue: if 50% of injected activity was present in normal thyroid tissue, tumour concentrations were doubled; if 75% was present, values were multiplied by four. Determination of minimal detectable activity showed that samples with a normalised iodine concentration of at least 5·10^−6^ IA·g^−1^ was adequately quantified with the setup used in the study.

### Histopathology work-up

All tumours underwent histopathological examination by an experienced endocrine pathologist. The 2022 WHO classification of endocrine tumours was followed [16]. PTCs were diagnosed based on nuclear features and growth pattern. For PDTCs, the so-called Turin criteria were used [17]. The slices were studied microscopically to verify the tissue representativity to avoid any confounding by high uptake in normal thyroid tissue. The pTNM staging was performed according to the AJCC version 8 criteria [18]. Histological subtyping, pTNM staging and immunohistochemistry based on pathological analysis on the initial surgery specimens were used throughout all analysis, regardless of any later re-staging.

### Analysis of initial and follow-up disease status

At the time of the first RAI treatment, laboratory data for Tg and Tg-Ab both under TSH suppression and TSH stimulation were collected. Post-therapeutic iodine-131 whole body scintigraphy (WBS) data was collected at 6–8 days post RAI after the first and any subsequent RAI treatments. All radiological scans included in the analysis were independently reviewed by a senior consultant radiologist and nuclear medicine physician to validate the clinical assessment of the images.

Follow-up visits were conducted according to the Swedish national guidelines for thyroid cancer [19]. These involve a once-yearly follow-up after surgery with more frequent visits for patients with advanced or persistent disease. Disease status during follow-up was assessed by clinical examination, evaluation of serum Tg and Tg-Ab levels (routinely both under TSH suppression and stimulation at the first-year visit) and when indicated, ultrasound and fine-needle aspiration cytology (FNAC). Diagnostic WBS, SPECT/CT (using iodine-123, combined with a diagnostic chest CT) and stand-alone CT were performed when considered clinically indicated.

### Assessment of persistent disease

Biochemical persistent disease was defined as elevated Tg levels after at least one RAI treatment, as serum Tg >0.1 ng/ml (suppressed) or >1 ng/ml (stimulated). If serum Tg was absent in combination with present Tg-Ab, the patient was scored as “no biochemical persistent disease” only if the Tg-Ab levels were decreasing over time. Patients were considered to have local persistent disease if structurally visible tumour was seen on ultrasound or CT images. Regional persistent disease was scored when ultrasound, FNAC, WBS, SPECT or CT showed evidence of lymph node metastases. Distant metastatic disease was defined as positive findings on WBS, SPECT or CT.

### Assessment of iodine avidity in persistent disease

The definition of iodine avidity was based upon guidelines from the American Thyroid Association and a review of RAI-refractory disease management by Jin et al. [3, 20]. While some controversy exists with regards to the definition of refractory disease [21], these guidelines were chosen to allow comparison to other publications based on the same criteria. Iodine avidity was assessed according to the following criteria (assessed by WBS and SPECT/CT):

(I) no concentration of RAI in metastatic foci

(II) the loss of RAI uptake in persisting foci

(III) concentration of RAI in only some persisting foci

(IV) progressive metastases within one year of treatment despite RAI uptake

Tumour-to-background (T/B) ratios were also calculated based on SPECT (when available) and WBS images acquired 6–8 days after initial RAI treatment on all patients with visible disease, using methodology described by Yang et al. and Meng et al. [22, 23]. The count-rate in lesions were compared to background signal in the brain.

### Statistical analysis

Statistical analysis was performed using R Version 3.6.3 (R-project.org). In cases where several samples were taken from the same patient of either primary tumour or lymph node metastases, the geometric mean of the iodine concentrations was calculated and used in the analysis. This was done since the data on iodine avidity was found to approximately follow a log-normal distribution, and because the geometric mean is robust to small sample sizes and estimates the median of the log-normal distribution well. Data on iodine avidity, Tg expression and Ki-67 was log-transformed prior to tests because of the approximate log-normal distribution of those datasets. Differences in iodine avidity between groups of patients were tested using unpaired Welch’s *t*-test. Differences in iodine avidity within the same patient were tested using paired Welch’s *t*-test. Correlations were calculated using Pearson’s product-moment correlation coefficient *ρ*.

### Ethical considerations

The study was approved by the Regional Ethical Review Board in Stockholm and the Radiation Protection Committee at Karolinska University Hospital (2017/2393-31, 2020-01222). Informed written consent was obtained from all patients before study inclusion. The individual radiation effective dose caused by study participation was low, estimated to between 2 and 3 mSv, which is less than 1% of the effective dose from a standard radioiodine treatment, and approximately equivalent to background radiation over two years.

## Results

Out of 45 included patients, 35 had reliable measurements of tumoural and metastatic iodine concentrations at the point of surgery. Ten patients were excluded, because of: incorrect cytological diagnosis (*n* = 4), primary tumour too small to examine (*n* = 3), too long delay between injection and surgery (*n* = 3). In total, 74 samples of primary tumour and metastases were collected (including multiple samples from the same patient). The median follow-up time was 31 months (range 19–46 months). Patient and sample characteristics are shown in Table 1.

**Table 1:** Characteristics of all patients, primary tumour and initial lymph node metastasis samples collected at initial surgery.

Parameter	Total (*n* = 35)			
Age at Inclusion	53 (19,81)			
Median (range)				
Sex				
Male	14 (40%)			
Female	21 (60%)			
Persistent Disease				
Yes	10 (29%)			
Radioiodine Treatment				
Yes	32 (91%)			
Initial Staging	**pT**		**pN**	
0/x	0		17	
1a	5		7	
1b	9		11	
2	13		-	
3a	3		-	
3b	5		-	
**Histological Subtype**	**Primary Tumour (*n*** = **38)**	**Lymph Node Metastasis (*n*** = **36)**		**Total (*n*** = **74)**
Conventional PTC	9 (24%)	9 (25%)		18 (24%)
Diffuse sclerosing PTC	4 (11%)	14 (39%)		18 (24%)
Warthin-like PTC	2 (5%)	0 (0%)		2 (3%)
Follicular subtype of PTC	2 (5%)	3 (8%)		5 (7%)
Oxyphilic PTC	2 (5%)	2 (6%)		4 (5%)
Hobnail PTC	2 (5%)	1 (3%)		3 (4%)
Tall-cell PTC	8 (21%)	7 (19%)		15 (20%)
Differentiated high-grade thyroid cancer	3 (8%)	0 (0%)		3 (4%)
Poorly differentiated thyroid cancer	6 (16%)	0 (0%)		6 (8%)

This summary includes multiples samples from the same patient, when available

### Iodine avidity in patients with persistent disease

**Fig. 1:**
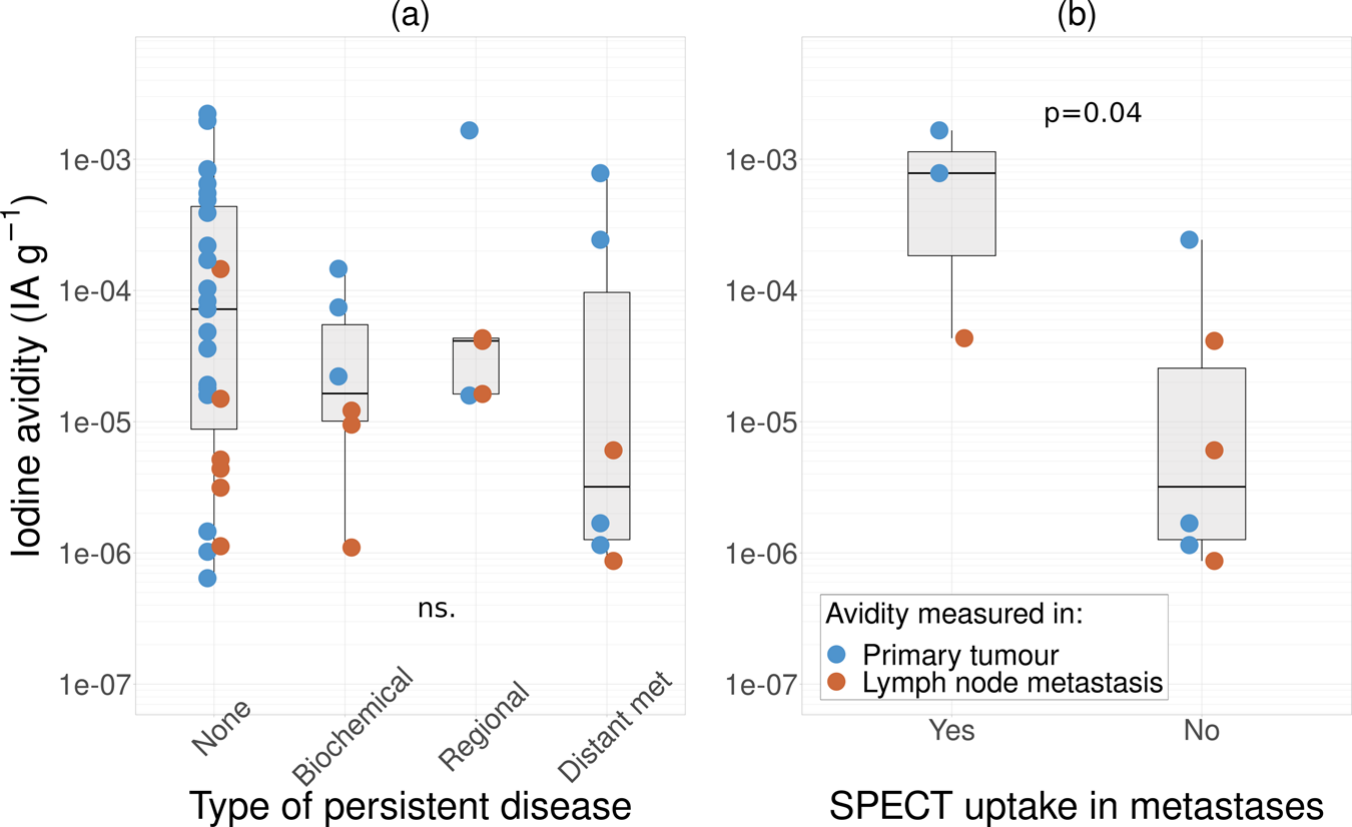
**a** Iodine avidity for tumour tissue in patients without or with biochemical, regional and distant metastatic persistent disease. **b** Iodine avidity for tumour tissue in patients with regional or distant metastases, and those without structurally detectable disease. Patients with structurally detectable disease were grouped depending on uptake status in metastases. Iodine avidity in both primary tumours and initial lymph node metastases are shown. NED no evidence of structural disease

Iodine avidity, quantified at surgery in primary tumours and lymph node metastases, for patients with and without persistent disease is shown in Fig. 1a. The iodine avidity measured at surgery was not significantly lower among those with persistent disease than in those without any signs of disease (2.3-fold for persistent disease, CI 0.54–10). The relation between pre-therapeutic iodine avidity versus iodine avidity in metastases for persistent disease is displayed in Fig. 1b. The results show that patients with non-avid persistent disease had a statistically significant 55-fold (CI 1.3–2400 fold) lower iodine avidity in primary and initially removed lymph node metastases than patients with avid persistent disease. A comparison of iodine avidity in primary tumours and lymph node metastases within and between patients is shown in Supplementary Information 1.

### Characteristics of patients with iodine-avid persistent disease

**Fig. 2:**
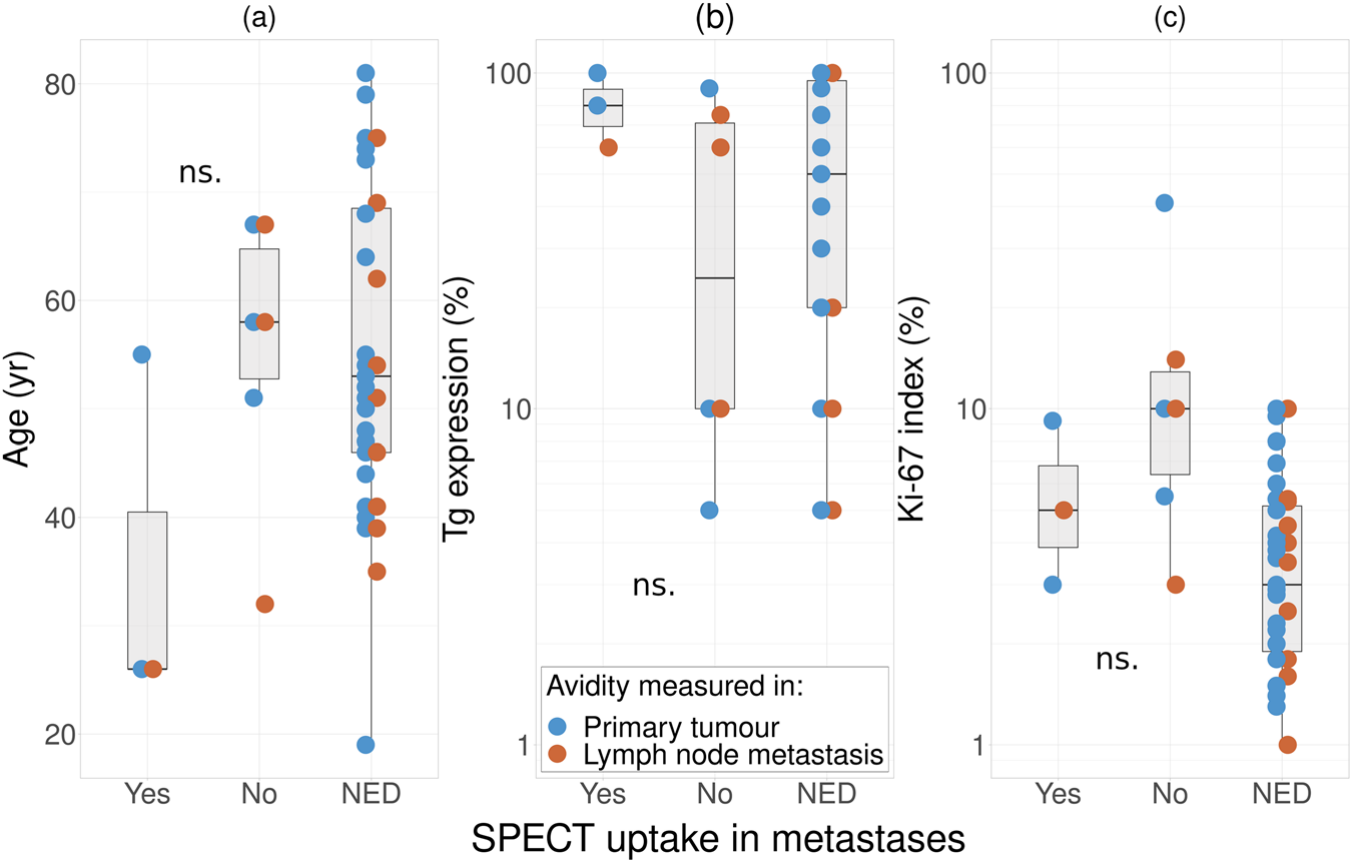
Relation between (**a**) patient age, (**b**) Tg expression and (**c**) Ki-67 index and iodine uptake in metastases. Data on Tg expression and Ki-67 index is presented for both primary tumour and initial lymph node metastases. NED no evidence of structural disease

All patients with iodine-avid metastases during follow-up were younger than 60 years, however with a large overlap between the groups, as displayed in Fig. 2a. There was no significant difference in age between patients with and without persistent disease. All patients with regional or distant metastases that were classified as iodine-avid had Tg expression in more than 75% of cells in their primary tumour and lymph node metastases at initial surgery, as shown in Fig. 2b. No significant difference was found between Tg expression in patients with and without persistent disease, nor between patients with iodine avid and non-avid metastases. Iodine avid persistent disease had Ki-67 indices below 10% at initial surgery, albeit with a overlap between the groups, as can be seen in Fig. 2c. No persistent disease was found in patients with Ki-67 < 3%. The geometric mean of Ki-67 indices was 3.1% in patients without persistent disease. Ki-67 indices was significantly higher in patients with persistent disease, by an average of 2.7 percentage points (CI 0.2–7.2 percentage points).

**Fig. 3:**
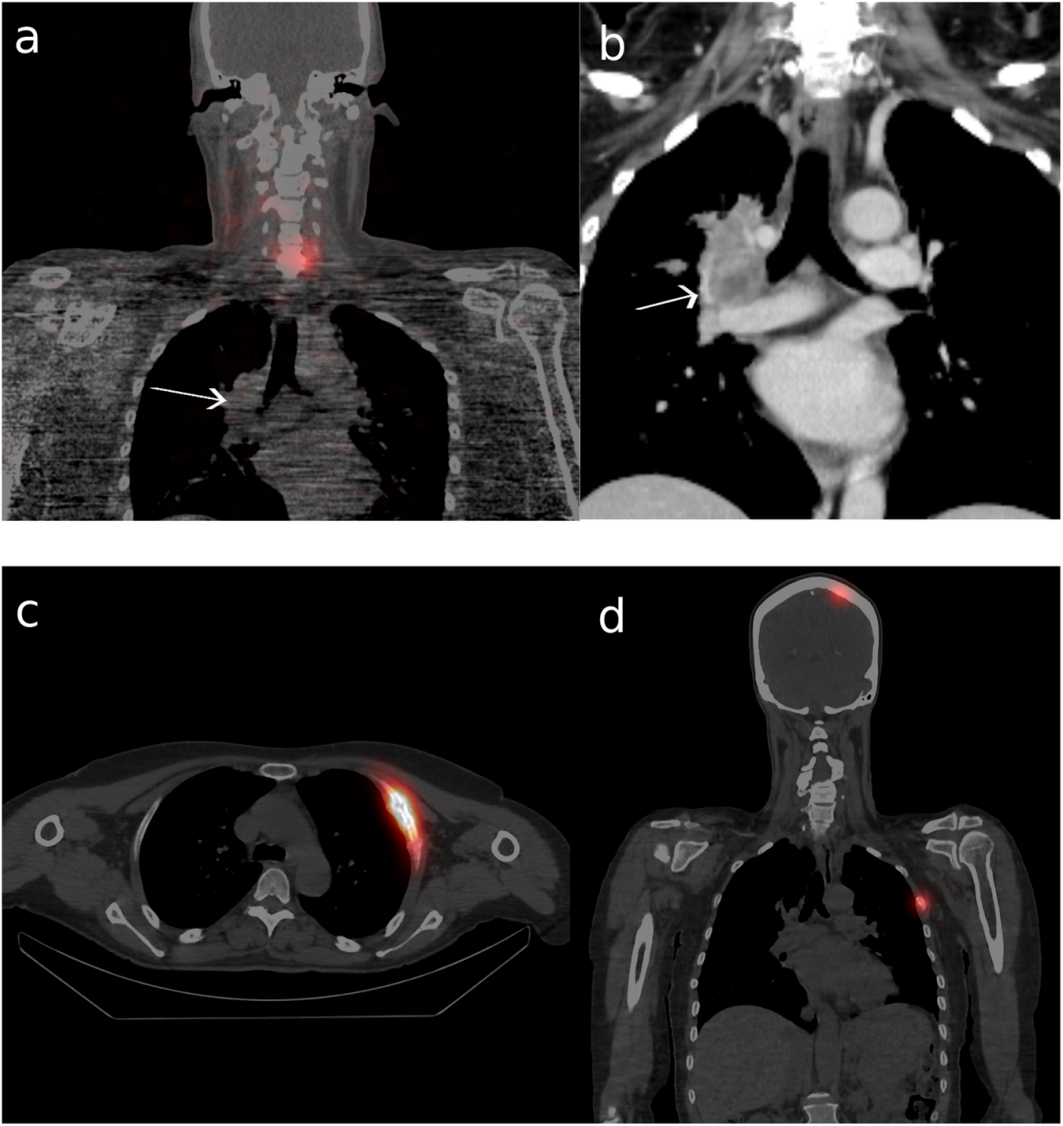
**a** SPECT/CT and (**b**) contrast-enhanced diagnostic CT image (taken 1.5 months after the SPECT/CT in (**a**)) of #2 showing a bronchial metastasis with no visible iodine uptake. Note iodine uptake in remnant tissue on the neck for reference. **c** Transversal and (**d**) coronal SPECT/CT image of #9, showing high iodine uptake in metastatic lesions in the skull and rib

Further details on characteristics of all the patients with persistent disease are shown in Table 2. Examples of iodine avidity assessment on SPECT/CT for two patients are shown in Fig. 3. Two patients died from distant metastases of their thyroid cancers, 18 and 17 months after their respective date of diagnosis. The primary tumour of #2 was initially inoperable; only after courses of external beam radiotherapy and tyrosine kinase inhibitor therapy was surgical resection and RAI treatment possible. The metastases in lymph nodes and lungs of #6 did not respond to RAI treatment and they continued progressing despite tyrosine kinase inhibitors. Results from dosimetric calculations performed outside study protocol in two patients are shown in Supplementary Information 2.

**Table 2:** All ten cases of persistent and recurrent disease. Follow-up duration measured from cytological diagnosis

ID	Sex/Age	Surgery	Macro/micro radic-ality	Histologi-cal subtype	pTNM	RAI activity 1st/2nd treatment [MBq]	Time to event after RAI [months]	Type of persistent disease	Iodine uptake in metastases (T/B-ratio)	Serum Tg pre/post first RAI	Follow-up duration [months]
#1	M51	Total + LN central/dx	y/y	oncocy-tic PTC	T2N1b	7400	24	biochemical	*(*)	0.3/0.4	35
#2	F51	Inoperable/total	n/n	PDTC	T3bNx	7400	0	local/ distant met	no (3)	251/587	18 †
#3	M75	Total + LN central/sin	n/y	cPTC	T3aN1b	5550	12	biochemical	*(*)	13/4.6	26
#4	F26	Total + LN central/ bilateral	y/y	dsPTC	T1bN1b	5550	4	regional	yes (1000)	19/6.4	23
#5	F46	Total + LN central	y/y	tcPTC	T2N1a	3700	9	regional	*(*)	0.3/0.6	22
#6	M67	Total + LN central	n/n	hPTC	T3bN1b	5550	7	regional/distant met	no (1)	not detectable /15	17 †
#7	M54	Total + LN central/ bilateral	y/y	cPTC	T2N1b	5550	9	biochemical	*(*)	3.1/4.1	19
#8	F32	Total + LN central/dx	y/y	dsPTC	T1bN1b	5550	0	regional	no (2)	not detectable/ not detectable	15
#9	F55	Total + LN central	y/y	PDTC	T3aN0	3700/7400	0	distant met	yes (500)	3200/1800	14
#10	M58	Total + LN central	n/n	tcPTC	T2N1a	5550	0	distant met	no (1)	47/153	13

*dsPTC* diffuse sclerosing subtype of PTC, *tcPTC* tall cell subtype of PTC, *hPTC* hobnail subtype of PTC, *cPTC* conventional subtype of PTC, *NA* not available, *T/B* tumour to background, *LN* lymph node dissection, *sin* left, *dx* right, † death, *not possible to assess due to no visible structural disease

In #5, a lymph node metastasis of 7 mm was detected during follow-up and was thereafter surgically removed. It could not be visualised on any nuclear imaging or CT and it was therefore not possible to assess avidity. #1, #3 and #7 had only biochemical signs of disease and were treated conservatively. Surgical radicality at primary operation was non-complete in three out of four patients with non-avid disease.

## Discussion

In this work on iodine avidity, we present a unique comparison between the pre-therapeutic tumoural avidity and the iodine uptake in metastases during follow-up (as positivity in subsequent metastases on WBS and SPECT/CT using either iodine-131 or iodine-123). They showed clear agreement, confirming that tumoural iodine concentration at surgery, even under euthyroid conditions, is a predictor of avidity in regional and distant metastases.

This method of measuring iodine concentrations in surgical specimens overcomes limitations of iodine avidity assessments based on WBS and SPECT, caused by limited sensitivity and spatial resolution. In other published studies assessing iodine avidity in lymph node or distant metastases, the WBS and SPECT methods are almost exclusively used to determine the binary classification avid/non-avid [23–25]. Two exceptions are studies that performed a semi-quantitative assessment of iodine avidity, normalising uptakes to background signal in WBS and SPECT image data, respectively [22, 23]. The current work was able to study the primary tumour avidity, which is not possible with WBS or SPECT, as normal thyroid signal would dominate and obscure any uptake in primary tumour tissue. Both binary and semi-quantitative approaches have found links between primary tumour *BRAF* and *TERT* promoter mutations and avidity in metastatic lesions [26, 27].

The pre-therapeutical data from both primary tumours and their respective lymph node metastases suggested that iodine avidity could differ between lesions within the same patient, but no significant difference was observed in this study, neither within the same patient nor between groups, see Supplementary Information 1. Collection of samples was done at a single time point (as it was the only feasible way). However, single time point proxies can estimate absorbed dose with satisfactory precision given that the time point is chosen with care, as has been shown in other publications [14, 15].

This work aimed to calculate tumour-to-background ratios in a similar way to results published by Yang et al. in the semi-quantitative approach [22]. The results were comparable, although the ratios for avid metastases in this work was higher than reported by others, possibly explained by later scan times (not reported by Yang et al.) and superior quantification by SPECT used in this work. Additionally, the tumour dosimetry performed on two patients in this work further confirmed the avidity status of lesions in those patients, shown in Supplementary Information 2.

The concordance of tumoural iodine concentration at surgery with image based methods of estimating iodine avidity, and the advantages compared to such methods, suggests it may be a useful tool in studying iodine avidity the future.

The patients in this cohort had a higher rate of persistent disease than expected in PTC overall, taking into account the short follow-up time [28]. This can in part be explained by the inclusion of PDTC, as 2 out of 3 patients with PDTC had persistent disease. The proportion of men, who more frequently have high-risk disease, was also higher compared to the general PTC population. While this introduces bias towards more aggressive disease, the aim of this work was to study the specific link of tumoural avidity to outcome, not the risk of persistent disease in the larger PTC population. Regardless, the present study is conducted in a smaller prospectively collected cohort, which may limit generalisation to the full PTC and PDTC populations.

Previous analysis of the patients in the same cohort as in this study showed significant correlation between both Ki-67 indices and thyroglobulin expression, and tumoural avidity [13]. It was further found that Ki-67 index and thyroglobulin expression outperformed tumour size and TNM staging as correlates to tumoural iodine avidity. In the present analysis, Ki-67 indices at diagnosis were significantly higher for patients with persistent disease. This is in agreement with previous studies that have found Ki-67 index linked to clinical progression and persistent disease [29–31]. The results in the current work showed trends for higher age, lower Tg expression and higher Ki-67 indices for non-avid persistent disease, but no significant differences were found.

Patients with non-avid or RAI-refractory disease have worse prognosis and an unmet need for other therapeutic options, as RAI therapy is not able to treat their tumours [32, 33]. The results in this study may help identifying a subset of patients that could be helped by more aggressive initial radioiodine treatment, with higher activities, possibly using dosimetry-guided treatment [34]. The results in this work indicate a strong link between persistent disease avidity and primary tumour avidity, which can be assessed by initial histological and molecular analyses [13]. This has the potential to also aid in patient selection for early redifferentiation therapy, where another therapeutic agent may restore or elevate the ability to accumulate iodine in cancer tissue.

The conclusion from these results should therefore not be that each individual patient should undergo the elaborate preparations and measurement of radioactivity at surgery as used in the current study, as it is logistically challenging and causes extra costs. Instead, the shown links between avidity at surgery and in persistent disease may be utilised in individualising future treatment.

Several studies of combined redifferentiation and RAI treatment schemes have been conducted in recent years [35–38]. These studies have been focused on RAI-refractory patients, who have received several RAI treatments and often other therapies. It can be argued that earlier redifferentiation therapy in combination with RAI treatment may produce better results. It is likely that the first RAI treatment will result in the greatest iodine concentration in a tumour, since effective half-lives are likely to decrease over courses of RAI therapy [39]. Therefore, an early redifferentiation therapy may have a greater chance of increasing absorbed doses to a level where complete response is possible. Identifying patients that will benefit from such an approach would be of outmost importance, if redifferentiation therapy becomes established.

A close link between iodine concentration in primary tumours with iodine avidity of subsequent metastases was observed. This suggests that knowledge on iodine avidity based on characteristics of surgical specimens from initial surgery, could offer opportunities to personalise radioiodine treatment and improve outcomes to a greater degree.

### Supplementary Information

Nilsson et al. Endocrine Supplementary Information 1

Nilsson et al. Endocrine Supplementary Information 2
