# Supplementary material for: Primary tumour iodine avidity in relation to uptake in persistent metastatic disease in papillary and poorly differentiated thyroid cancer
Source: Endocrine. 2023 Jun 7;82(2):343–52. doi: 10.1007/s12020-023-03414-7 (PMC10543945; doi:10.1007/s12020-023-03414-7)
Supplement: Supplementary file 1 — Nilsson et al. Endocrine Supplementary Information 1 [file 12020_2023_3414_MOESM1_ESM.pdf]

Supplementary Information 1 - Comparison iodine avidity in primary tumours and synchronous lymph node metastases

Primary tumour iodine avidity in relation to uptake in persistent metastatic disease in papillary and poorly differentiated thyroid cancer
Endocrine

Joachim N. Nilsson*, Per Grybäck, C. Christofer Juhlin, Christel Hedman, Catharina Ihre Lundgren
*Department of Molecular Medicine and Surgery, Karolinska Institutet, joachim.nilsson@ki.se

Methods

Iodine concentrations measured in primary tumours and corresponding initial lymph node metastases were compared. The comparison was made in patients that had both primary tumours and macroscopically detectable lymph node metastases at pathology grossing. If available, multiple samples were taken from the same tissue type in the same patient; the geometric mean of measured concentrations was then used for those patients in the comparison.

Results

Out of the 17 patients that had lymph node metastases, we were able to collect tissue from both primary tumour and lymph node metastases at surgery in 10 patients; the iodine avidity of each sample is shown in Figure S1. Seven patients had lower iodine avidity in lymph node metastases than in primary tumours, one patient had similar values, and two patients had higher avidity in lymph node metastases. While the mean iodine avidity in primary tumour tissue compared to initial lymph node metastases was 5.7-fold higher, a paired *t*-test showed a borderline non-significant fold-difference (CI 0.90 - 36).

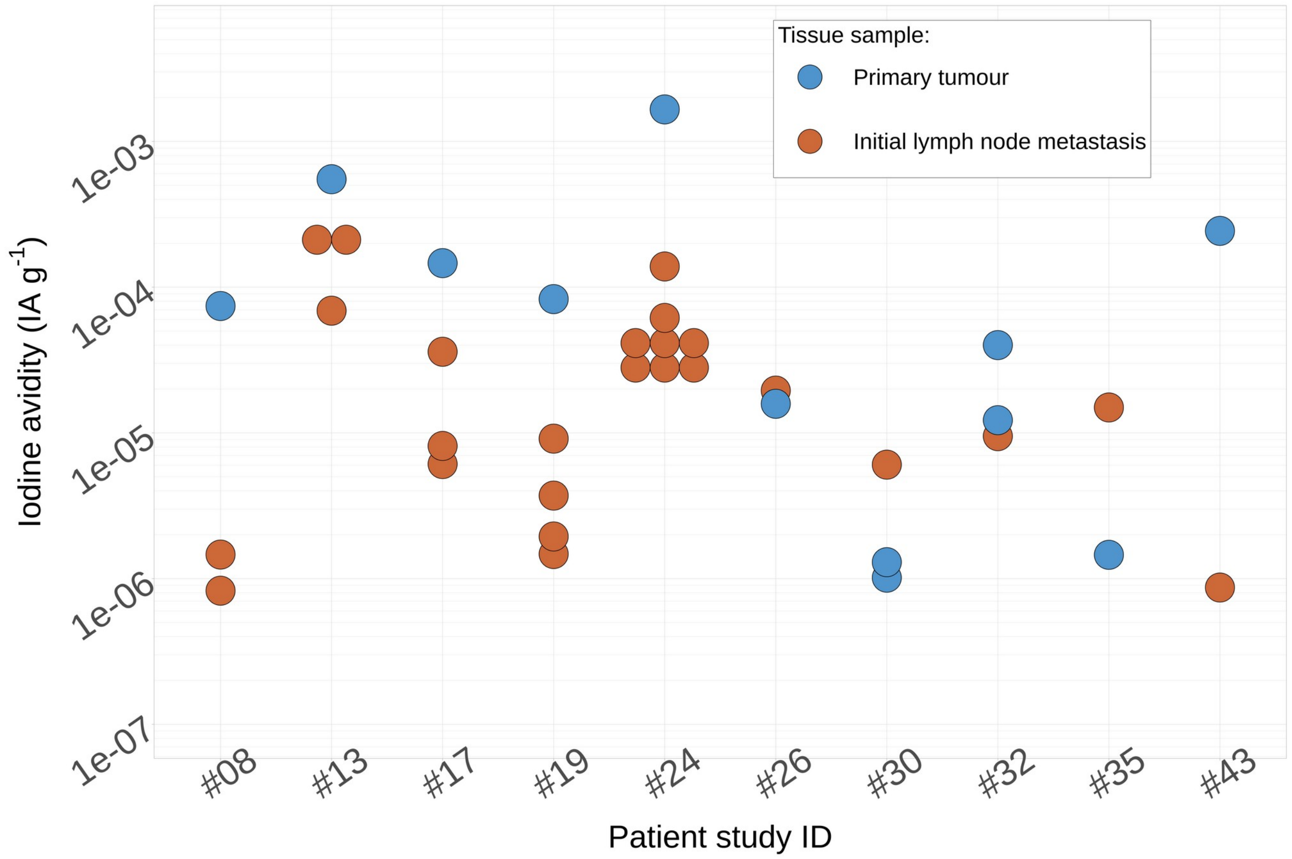

Figure S1: Iodine avidity in samples of primary tumour and initial lymph node metastases in the patients that had both removed during surgery. Patient study IDs are shown on the x-axis for reference.
