# Supplementary material for: Primary tumour iodine avidity in relation to uptake in persistent metastatic disease in papillary and poorly differentiated thyroid cancer
Source: Endocrine. 2023 Jun 7;82(2):343–52. doi: 10.1007/s12020-023-03414-7 (PMC10543945; doi:10.1007/s12020-023-03414-7)
Supplement: Supplementary file 2 — Nilsson et al. Endocrine Supplementary Information 2 [file 12020_2023_3414_MOESM2_ESM.pdf]

Supplementary Information 2 - Dosimetry supplement

Primary tumour iodine avidity in relation to uptake in persistent metastatic disease in papillary and poorly differentiated thyroid cancer
Endocrine

Joachim N. Nilsson*, Per Grybäck, C. Christofer Juhlin, Christel Hedman, Catharina Ihre Lundgren
*Department of Molecular Medicine and Surgery, Karolinska Institutet, joachim.nilsson@ki.se

Methods

Lesional dosimetry was performed when deemed clinically relevant, but was not part of the study protocol. Dosimetry was performed by dose kernel-convolution on SPECT data acquired at three time points. Targets were delineated by a radiologist based primarily on CT data. The time-activity curves were integrated as mono-exponentials. Activity concentrations in targets were corrected with recovery coefficients, established for each SPECT/CT-system by standard phantom measurements.

Results

In two patients (#2 and #9 in Table 2), post-therapeutic dosimetry was conducted to calculate absorbed doses to metastatic lesions. A lesion in the bronchus of #2 received less than 5 Gy from an administration of 7.4 GBq iodine-131 and was classified as non-avid; radiology of the metastasis is shown in Figure 4a and 4b. In #9, skeletal metastases received cumulated absorbed doses of more than 100 Gy from two administrations of a total of 11.1 GBq iodine-131 and the metastases were classified as iodine avid, see Figure 3c and 3d.

These results were in line with high/low avidity on images and with the tumour-to-background ratio.
